# Re-immunotherapy with nivolumab plus ipilimumab in advanced non-small cell lung cancer patients previously treated with anti-programmed death-1 and/or anti-programmed death ligand-1 antibodies

**DOI:** 10.1007/s12672-023-00781-5

**Published:** 2023-08-31

**Authors:** Takuma Imakita, Kohei Fujita, Takanori Ito, Zentaro Saito, Issei Oi, Osamu Kanai, Hiromasa Tachibana, Satoru Sawai, Tadashi Mio

**Affiliations:** 1 Division of Respiratory Medicine, Center for Respiratory Diseases, National Hospital Organization Kyoto Medical Center, 1-1, Fukakusa-Mukaihata, Fushimi, Kyoto, 612-8555 Japan; 2 Division of Thoracic Surgery, Center for Respiratory Diseases, National Hospital Organization, Kyoto Medical Center, 1-1, Fukakusa-Mukaihata, Fushimi, Kyoto, 612-8555 Japan

**Keywords:** Cytotoxic T-lymphocyte-associated protein-4, Tumor proportion score, Immune checkpoint inhibitor, Immune-related adverse event, Objective response rate

## Abstract

**Background:**

The role of re-immunotherapy in advanced non-small cell lung cancer (NSCLC) remains unclear. No studies have evaluated the re-immunotherapy regimen including anti-cytotoxic T-lymphocyte antigen-4 antibody for lung cancer treatment. This study aimed to investigate the efficacy and safety of re-immunotherapy with nivolumab plus ipilimumab in patients with advanced NSCLC previously treated with anti-programmed death-1 (PD-1) and/or anti-programmed death ligand-1 (PD-L1) antibodies.

**Methods:**

We retrospectively reviewed patients with advanced or recurrent NSCLC who received immunotherapy with nivolumab plus ipilimumab (without concomitant cytotoxic chemotherapy) between November 2020 and November 2022 at the National Hospital Organization Kyoto Medical Center, Kyoto, Japan. Data were extracted from patients who had previously received immunotherapies with anti-PD-1 and/or anti-PD-L1 antibodies. Treatment responses and adverse events were evaluated.

**Results:**

Of the 67 patients who received immunotherapy with nivolumab plus ipilimumab, 23 were included in final analysis. The objective response rate was 17%, and the disease control rate was 48% for nivolumab plus ipilimumab therapy. The highest grade of immune-related adverse events was grade 3, occurring in 11% of cases.

**Conclusion:**

Re-immunotherapy with nivolumab plus ipilimumab after anti-PD-1 and/or anti-PD-L1 immunotherapy may be feasible and provide clinical benefit in selected patients. Further prospective studies are warranted to identify the patient population that may benefit from re-immunotherapy.

## Introduction

Immunotherapy with immune checkpoint inhibitors (ICIs) has dramatically changed treatment strategies for advanced non-small cell lung cancer (NSCLC) [1–5]. The first-line treatment has shifted from platinum-doublet chemotherapy to immunotherapy with or without cytotoxic chemotherapy [6–8]. In this era of immunotherapy, previous evidence for second- or later-line treatment also needs to be reconsidered. The standard of care after immunotherapy failure is cytotoxic chemotherapy; however, the role of re-immunotherapy remains unclear. We have previously evaluated re-immunotherapy with anti-programmed cell death protein-1 (PD-1) antibody after anti-PD-1 antibody [9], anti-programmed cell death ligand-1 (PD-L1) antibody after anti-PD-1 antibody [10], and anti-PD-1 antibody after anti-PD-L1 antibody [11]. However, to our knowledge, only for the treatment of melanoma and not for the treatment of lung cancer have studies evaluated re-immunotherapy with anti-cytotoxic T-lymphocyte antigen-4 (CTLA-4) antibody after immunotherapy with anti-PD-(L)1 antibody [12].

Anti-CTLA-4 antibody potentially exerts a different mechanism of action in the cancer-immunity cycle than anti-PD-(L)1 antibodies [13]. CTLA-4, expressed on T cells, interacts with its ligands (B7.1 and B7.2) on antigen-presenting cells to mediate signals that inhibit T cell priming and activation in lymphoid tissues. PD-L1 expressed on the tumor cell surface binds to PD-1 on effector T cells and inhibits anti-tumor cytotoxic T cell responses in the tumor microenvironment [13–15]. Therefore, CTLA-4-targeted immunotherapy may be effective in patients who are refractory to anti-PD-(L)1 immunotherapy.

This study aimed to investigate the efficacy and safety of re-immunotherapy with nivolumab (anti-PD-1 antibody) plus ipilimumab (anti-CTLA-4 antibody) in patients with advanced NSCLC previously treated with anti-PD-1 and/or anti-PD-L1 antibodies.

## Methods

This retrospective cohort study was conducted at the National Hospital Organization Kyoto Medical Center, Kyoto, Japan. We reviewed patients with advanced or recurrent NSCLC who received immunotherapy with nivolumab and ipilimumab between November 2020 and November 2022. The inclusion criteria were as follows: (i) pathologically confirmed NSCLC, (ii) with advanced stage, unresectable disease, or postoperative recurrence, (iii) administration of at least one dose of nivolumab (360 mg intravenously every 3 weeks or longer) and ipilimumab (1 mg/kg intravenously every 6 weeks or longer), and (iv) previous immunotherapy with anti-PD-1 and/or anti-PD-L1 antibodies. During this study, nivolumab and pembrolizumab were administered as anti-PD-1 antibodies and atezolizumab and durvalumab were administered as anti-PD-L1 antibodies. Patients who received durvalumab were excluded from this study, which focused on immunotherapy for advanced NSCLC. This is because durvalumab was only approved for maintenance therapy after concurrent chemoradiotherapy for locally advanced NSCLC in Japan. Patients who received combination chemo-immunotherapy with nivolumab, ipilimumab and cytotoxic anticancer agents were also excluded. Data extracted from the clinical records included patient characteristics (age, sex, body mass index, smoking history, Eastern Cooperative Oncology Group [ECOG] performance status [PS], histological type of cancer, clinical stage, driver oncogene alterations, and tumor proportion score [TPS] of PD-L1) and treatment profiles (regimen, response, time to treatment failure [TTF], immune-related adverse events [irAEs], and overall survival). Treatment response was evaluated based on the Response Evaluation Criteria in Solid Tumors version 1.1 [16]. Adverse events were graded according to the Common Terminology Criteria for Adverse Events version 5.0 [17]. The observational period was from November 2020 to January 2023. The study protocol was approved by the Ethical Committee and the Institutional Review Board of the National Hospital Organization Kyoto Medical Center.

## Results

### Patients’ characteristics

**Fig. 1:**
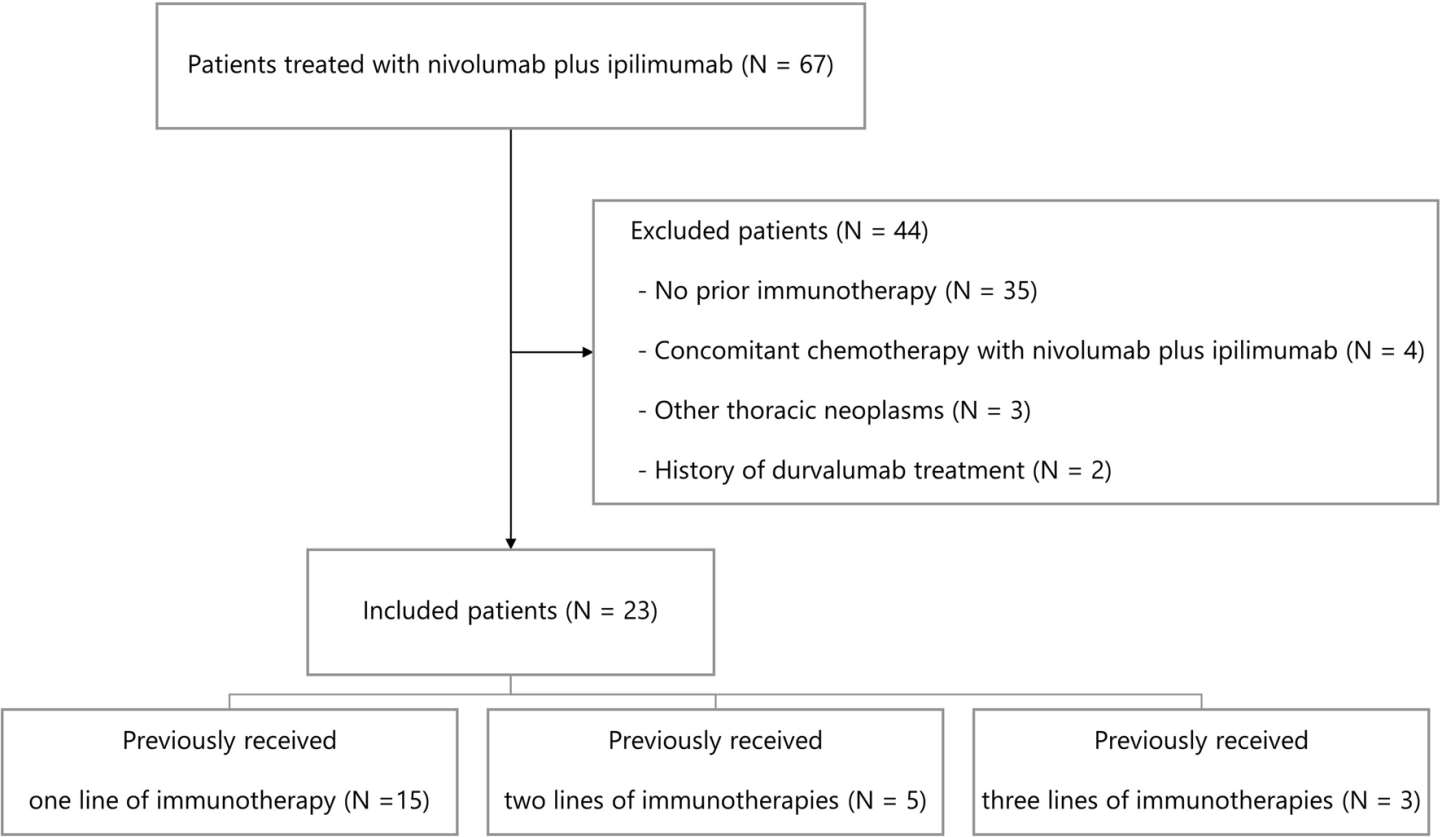
Flow chart of study populations

Between November 2020 and November 2022, 67 patients received immunotherapy with nivolumab and ipilimumab. Among these patients, 44 were excluded (35 had no history of prior immunotherapy, 4 received concomitant cytotoxic chemotherapy with nivolumab and ipilimumab, 3 were treated for other thoracic neoplasms, and 2 had history of durvalumab maintenance therapy after chemoradiotherapy). The remaining 23 were included for further analyses. The flow chart of study populations is shown in Fig. 1.

The baseline characteristics of the patients are summarized in Table 1. Overall, 18 (78%) patients were male. The median age at the time of nivolumab plus ipilimumab therapy was 72 (range, 59–84) years, and eight (35%) patients were 75 years or older. Sixteen (70%) patients had adenocarcinoma, of which four patients harbored mutations in the epidermal growth factor receptor gene. Fourteen (61%) patients had PD-L1 expression in at least 1% of tumor cells. In addition, 18 (78%) patients had an ECOG-PS score of 0 or 1.

**Table 1:** Patients’ characteristics

	N = 23^1^
Sex	
Male	18 (78%)
Female	5 (22%)
Age	72 [59–84]
≥ 75 years	8 (35%)
BMI	22.1 ± 3.5
Smoking history	
Current	8 (35%)
Former	11 (48%)
Never	4 (17%)
ECOG-PS	
0	5 (22%)
1	13 (57%)
2	4 (17%)
3	1 (4%)
Histology	
Adenocarcinoma	16 (70%)
Squamous cell carcinoma	6 (26%)
NSCC, NOS	1 (4%)
Stage at diagnosis	
I	2 (9%)
II	1 (4%)
III	7 (30%)
IV	13 (57%)
Postoperative recurrent	5 (22%)
PD-L1 TPS	
< 1%	7 (30%)
1–49%	10 (43%)
50%≦	4 (17%)
Unknown	2 (9%)
Driver mutation	
None	19 (83%)
EGFR exon20 insertion	1 (4%)
EGFR L861Q	1 (4%)
EGFR L858R	1 (4%)
EGFR L858R, T790M	1 (4%)

*BMI* body mass index, *ECOG* Eastern Cooperative Oncology Group, *PS* performance status, *NSCC* non-small cell cancer, *NOS* not otherwise specified, *PD-L1* programmed death ligand-1, *TPS* tumor proportion score, *EGFR* epidermal growth factor receptor

^1^n (%); median [range]; mean ± standard deviation

### Profiles of prior immunotherapy

Of the 23 patients, eight received two lines of immunotherapies, and three received three lines of immunotherapies prior to treatment with nivolumab plus ipilimumab (Fig. 1). Table 2 summarizes the treatment profiles based on the lines of prior immunotherapy. The objective response rate (ORR) in each treatment line was 48%, 38%, and 0%, respectively. As the treatment progressed, the median TTF became shorter at 7.6, 5.9, and 3.0 months. Severe irAEs (grade ≥ 3) were observed in 21%, 25%, and 33% of patients in each line, respectively. The most common reason for immunotherapy cessation was progressive disease during the first or second immunotherapy.

**Table 2:** Profiles of previous immunotherapy

	1st immunotherapy N = 23^1^	2nd immunotherapy N = 8^1^	3rd immunotherapy N = 3^1^
ICI			
Atezolizumab	4 (17%)	3 (38%)	1 (33%)
Nivolumab	1 (4%)	3 (38%)	2 (67%)
Pembrolizumab	18 (78%)	2 (25%)	0 (0%)
TTF (months)	7.6 [0.4–23.3]	5.9 [2.8–14.2]	3.0 [1.8–3.9]
Best response			
CR	0 (0%)	0 (0%)	0 (0%)
PR	11 (48%)	3 (38%)	0 (0%)
SD	12 (52%)	3 (38%)	2 (67%)
PD	0 (0%)	2 (25%)	1 (33%)
ORR (%)	48	38	0
DCR (%)	100	75	67
Highest grade of irAE			
Grade 0	14 (61%)	5 (62%)	1 (33%)
Grade 1	1 (4%)	1 (12%)	1 (33%)
Grade 2	3 (13%)	0 (0%)	0 (0%)
Grade 3	4 (17%)	2 (25%)	1 (33%)
Grade 4	1 (4%)	0 (0%)	0 (0%)
Reason for discontinuation			
PD	19 (83%)	7 (88%)	1 (33%)
irAE	3 (13%)	1 (12%)	2 (67%)
AE (other than irAEs)	1 (4%)	0 (0%)	0 (0%)

*ICI* immune checkpoint inhibitor, *TTF* time to treatment failure, *CR* complete response, *PR* partial response, *SD* stable disease, *PD* progressive disease, *ORR* objective response rate, *DCR* disease control rate, *irAE* immune-related adverse event, *AE* adverse event

^1^n (%); median [range]

### Profiles of immunotherapy with nivolumab plus ipilimumab

**Fig. 2:**
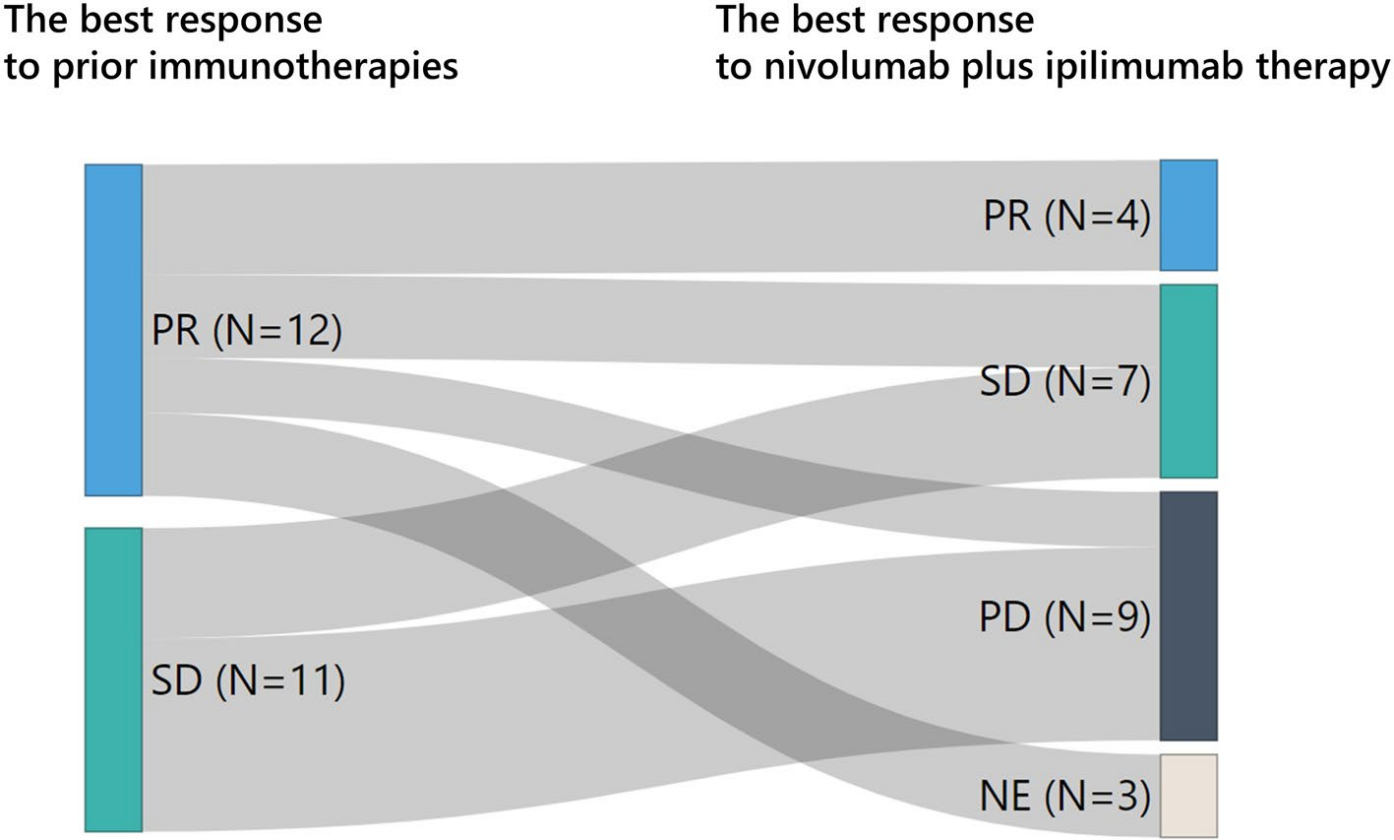
The relationships between the best response to previous immunotherapies and that to nivolumab plus ipilimumab therapy. *CR* complete response, *PR* partial response, *SD* stable disease, *PD* progressive disease, *NE* not evaluated

Table 3 describes the profile of re-immunotherapy with nivolumab plus ipilimumab. Median follow-up after starting nivolumab plus ipilimumab treatment was 5.9 (range, 0.5–22.2) months. The median TTF was 2.8 (range, 0.5–15.4) months. Four patients achieved partial response, and seven achieved stable disease. The ORR was 17%, and the disease control rate (DCR) was 48%. The reasons for treatment discontinuation were progressive disease (39%), adverse events (30%), and clinical decisions (e.g., worsened PS) (30%). The relationship between the best response to previous immunotherapies and nivolumab plus ipilimumab therapy is shown in Fig. 2. Among the four patients who achieved a partial response to nivolumab and ipilimumab therapy, the best response to previous immunotherapy (pembrolizumab monotherapy) was also a partial response, and their reason for treatment discontinuation was disease progression.

**Table 3:** Profiles of immunotherapy with nivolumab plus ipilimumab

	N = 23^1^
TTF (months)	2.8 [0.5–15.4]
OS (months)	8.2 [0.5–NR]
Best response	
CR	0 (0%)
PR	4 (17%)
SD	7 (30%)
PD	9 (39%)
NE	3 (13%)
ORR (%)	17
DCR (%)	48
Highest grade of irAE	
Grade 1	6 (22%)
Grade 2	10 (37%)
Grade 3	3 (11%)
Grade 4	0 (0%)
Reason for discontinuation	
PD	9 (39%)
irAE	7 (30%)
Clinical decision	7 (30%)

*TTF* time to treatment failure, *OS* overall survival, *NR* not reached, *CR* complete response, *PR* partial response, *SD* stable disease, *PD* progressive disease, *NE* not evaluated, *ORR* objective response rate, *DCR* disease control rate, *irAE* immune-related adverse events

^1^median [range]; n (%)

### Profiles of immune-related adverse events

The irAEs and their occurrence during nivolumab and ipilimumab therapy and previous immunotherapies are summarized in Table 4. In total, 34 regimens were used for previous immunotherapies. Colitis and rash were more frequently observed during nivolumab plus ipilimumab therapy than during previous immunotherapy, most of which were not severe (grades 1 or 2). The incidence of severe irAEs (grade ≥ 3) was lower during nivolumab plus ipilimumab therapy (n = 3, 13%) than during previous immunotherapy (n = 10, 29%). All severe irAEs were grade 3, except for one that was a grade 4 adverse event (platelet count decreased during atezolizumab treatment).

**Table 4:** Profiles of immune-related adverse events

	Previous immunotherapy (N = 34^1^)		Nivolumab plus ipilimumab therapy (N = 23^1^)	
	Grade ≤ 2	Grade ≥ 3	Grade ≤ 2	Grade ≥ 3
Adrenal insufficiency	0 (0%)	2 (6%)	1 (4%)	0 (0%)
Colitis	1 (3%)	1 (3%)	4 (17%)	1 (4%)
Hepatotoxicity	1 (3%)	2 (6%)	2 (9%)	0 (0%)
Hypothyroidism	2 (6%)	0 (0%)	1 (4%)	0 (0%)
Peripheral nerve disorder	0 (0%)	0 (0%)	1 (4%)	0 (0%)
Platelet count decreased	1 (3%)	1 (3%)	0 (0%)	0 (0%)
Pneumonitis	0 (0%)	3 (9%)	1 (4%)	2 (9%)
Rash	1 (3%)	1 (3%)	4 (17%)	0 (0%)

^1^n (%)

## Discussion

To our knowledge, this is the first report to evaluate the efficacy and safety of re-immunotherapy with nivolumab plus ipilimumab for patients with advanced NSCLC previously treated with anti-PD-1 and/or anti-PD-L1 antibodies. In this study, disease control was achieved in 11 (48%) of 23 patients during re-immunotherapy with nivolumab plus ipilimumab, including four (17%) patients who achieved a partial response. Previous retrospective studies showed that the efficacy of PD-(L)1-targeted re-immunotherapy after anti-PD-(L)1 therapy was limited (ORR 0–8.3%) [9–11]. Additionally, nivolumab retreatment, even for patients who responded to prior immunotherapy, showed a limited effect in a prospective study (ORR 8.5%) [18]. This limited efficacy may be due to the acquisition of resistance [19] and a physically or immunologically exhausted status with late-line therapy [10]. In this study, the response to nivolumab plus ipilimumab therapy appears to be superior to those reported for anti-PD-(L)1 re-immunotherapy; however, the small number of cases and retrospective nature of the study makes rigorous comparisons difficult. On the other hand, the efficacy of prior anti-PD-(L)1 immunotherapies decreased with each repeated treatment (Table 2), which is consistent with previous reports.

The target population for re-immunotherapy has not been established. However, previous reports on anti-PD-(L)1 antibody rechallenge indicate certain populations for re-immunotherapy. First, the efficacy may differ based on the combination of prior and rechallenged ICIs. Patients who received the same type of ICI as before (e.g., anti-PD-1 antibody after anti-PD-1 antibody) tended to maintain equivalent efficacy, while those rechallenged by different types of ICI (e.g., anti-PD-L1 antibody after anti-PD-1 antibody) showed a decreased response [20]. Similarly, for ipilimumab, which is a different type of ICI (anti-CTLA-4 antibody) from the previous ones (anti-PD-1 and/or anti-PD-L1 antibodies), the ORR for nivolumab plus ipilimumab immunotherapy was lower than that of the prior immunotherapy, and the best response was equal or inferior to that of the prior immunotherapy (as shown in Fig. 2). Four patients who achieved a partial response during nivolumab plus ipilimumab therapy also responded to prior immunotherapy. Further, none of the patients who did not respond to previous immunotherapy responded to nivolumab plus ipilimumab treatment. These results suggest that re-immunotherapy with anti-CTLA-4 antibody may not benefit patients unless the prior immunotherapy with anti-PD-(L)1 antibody was effective.

Second, reasons for discontinuation of immunotherapy may be related to the efficacy of the treatment. Haratani et al. reported that patients who developed irAEs achieved a longer PFS than those who did not [21]. This tendency was also observed in rechallenge situations. Some studies have reported that ICI retreatment results in a favorable response after irAEs in patients with NSCLC [22–24]. According to a systematic review evaluating solid tumors, the ORR to ICI rechallenge after development of toxicity was superior to that after disease progression (44% vs. 15.2%) [25]. Another systematic review of advanced NSCLCs revealed that the ORR and DCR of ICI retreatment were 20% and 54%, respectively [20]. However, these relatively high responses were probably due to the heterogeneity of the included studies, and the responses were improved in cases where re-administration occurred after irAEs. In our study, all four patients who responded to nivolumab plus ipilimumab therapy had discontinued prior immunotherapy because of disease progression. A possible explanation for this is that anti-CTLA-4 antibody suppresses cancer immunity through a mechanism different from anti-PD-1 or anti-PD-L1 antibodies by priming and activating tumor-specific T cells [13–15]; hence, it may affect tumors that have acquired resistance to PD-(L)1-targeted immunotherapy. Additionally, anti-CTLA-4 therapy combined with an anti-PD-1 antibody has a positive effect on PD-L1 negative NSCLC [5], while anti-PD-1 monotherapy has demonstrated lower efficacy [26, 27]. In our study, of the four cases that responded to re-immunotherapy, two were PD-L1 TPS negative; one had a TPS of 1–49%, and the other ≥ 50%, with no obvious trend identified in PD-L1 TPS.

The safety of re-immunotherapy following the development of irAEs is also a major clinical concern. Clinical practice guidelines recommend the permanent discontinuation of ICI after developing severe irAEs (mostly grade 4), although this depends on the type of irAE [28, 29]. A systematic review by Zhao et al. revealed a higher incidence of all-grade irAEs, and the incidence of severe irAEs was similar after ICI rechallenge [30]. In our study, the incidence of severe irAEs with nivolumab plus ipilimumab was lower than with previous immunotherapies, with no grade 4 irAEs (Tables 2, 3). However, 30% of patients discontinued immunotherapy due to irAEs (Table 3). Re-immunotherapy after non-severe and well-controlled irAEs may be feasible; nonetheless, physicians need to select patients carefully.

Our study had some limitations. It was retrospective and performed at a single facility; hence, it enrolled a small number of patients, and there was a possible selection bias. All treatment regimens were determined by the attending physicians and were not standardized between indivisuals. The small sample size did not allow for statistical analysis. Moreover, we evaluated only PD-L1 expression in tumors in clinical practice, and the expression of other immune checkpoint biomarkers (e.g., PD-L2, B7.1) were unknown. This study is at a preliminary stage, and we suggest that a larger prospective study be conducted to further investigate these findings.

## Conclusion

In this retrospective study, re-immunotherapy with nivolumab plus ipilimumab after anti-PD-1 and/or anti-PD-L1 immunotherapy had a higher ORR than PD-(L)1-targeted re-immunotherapy reported in previous studies. IrAEs were more frequently observed than in previous immunotherapies; nevertheless, most were not severe. Our results suggest that re-immunotherapy with anti-PD-1 and anti-CTLA-4 antibodies may be feasible and provide clinical benefit in selected patients. However, further prospective studies are required to identify the patient population that may benefit from re-immunotherapy.

## Data Availability

The datasets generated during and/or analysed during the current study are available from the corresponding author on reasonable request.

## Abbreviations

ICI: Immune checkpoint inhibitor
NSCLC: Non-small cell lung cancer
PD-1: Programmed cell death protein-1
PD-L1: Programmed death ligand-1
CTLA-4: Cytotoxic T-lymphocyte antigen-4
ECOG: Eastern cooperative oncology group
PS: Performance status
TPS: Tumor proportion score
TTF: Time to treatment failure
irAE: Immune-related adverse event
ORR: Objective response rate
DCR: Disease control rate
PFS: Progression-free survival

## References

[1] De Castro G, Kudaba I, Wu YL (2023). Five-year outcomes with pembrolizumab versus chemotherapy as first-line therapy in patients with non–small-cell lung cancer and programmed death ligand-1 tumor proportion score ≥ 1% in the KEYNOTE-042 Study. J Clin Oncol.

[2] Hellmann MD, Paz-Ares L, Bernabe Caro R (2019). Nivolumab plus ipilimumab in advanced non–small-cell lung cancer. N Engl J Med.

[3] Johnson ML, Cho BC, Luft A (2023). durvalumab with or without tremelimumab in combination with chemotherapy as first-line therapy for metastatic non–small-cell lung cancer: the phase III POSEIDON study. J Clin Oncol.

[4] Reck M, Mok TSK, Nishio M (2019). Atezolizumab plus bevacizumab and chemotherapy in non-small-cell lung cancer (IMpower150): key subgroup analyses of patients with EGFR mutations or baseline liver metastases in a randomised, open-label phase 3 trial. Lancet Respir Med.

[5] Paz-Ares LG, Ramalingam SS, Ciuleanu TE (2022). First-line nivolumab plus ipilimumab in advanced NSCLC: 4-year outcomes from the randomized, open-label, phase 3 checkmate 227 part 1 trial. J Thorac Oncol.

[6] Singh N, Temin S, Baker S (2022). Therapy for stage IV non-small-cell lung cancer without driver alterations: ASCO living guideline. J Clin Oncol.

[7] Planchard D, Popat S, Kerr K (2018). Metastatic non-small cell lung cancer: ESMO clinical practice guidelines for diagnosis, treatment and follow-up. Ann Oncol.

[8] Wu YL, Planchard D, Lu S (2019). Pan-Asian adapted clinical practice guidelines for the management of patients with metastatic non-small-cell lung cancer: a CSCO–ESMO initiative endorsed by JSMO, KSMO, MOS, SSO and TOS. Ann Oncol.

[9] Fujita K, Uchida N, Kanai O, Okamura M, Nakatani K, Mio T (2018). Retreatment with pembrolizumab in advanced non-small cell lung cancer patients previously treated with nivolumab: emerging reports of 12 cases. Cancer Chemother Pharmacol.

[10] Fujita K, Uchida N, Yamamoto Y (2019). Retreatment with anti-PD-L1 antibody in advanced non-small cell lung cancer previously treated with anti-PD-1 antibodies. Anticancer Res.

[11] Fujita K, Yamamoto Y, Kanai O (2020). Retreatment with anti-PD-1 antibody in non-small cell lung cancer patients previously treated with anti-PD-L1 antibody. Thorac Cancer.

[12] Aya F, Gaba L, Victoria I (2016). Ipilimumab after progression on anti-PD-1 treatment in advanced melanoma. Future Oncol.

[13] Chen DS, Mellman I (2013). Oncology meets immunology: the cancer-immunity cycle. Immunity.

[14] Wei SC, Duffy CR, Allison JP (2018). Fundamental mechanisms of immune checkpoint blockade therapy. Cancer Discov.

[15] Ribas A, Wolchok JD (2018). Cancer immunotherapy using checkpoint blockade. Science.

[16] Eisenhauer EA, Therasse P, Bogaerts J (2009). New response evaluation criteria in solid tumours: revised RECIST guideline (version 1.1). Eur J Cancer.

[17] Common Terminology Criteria for Adverse Events (CTCAE) v5.0. Cancer Therapy Evaluation Program (CTEP). Accessed March 3, 2023. https://ctep.cancer.gov/protocoldevelopment/electronic_applications/ctc.htm#ctc_50

[18] Akamatsu H, Teraoka S, Takamori S (2022). Nivolumab retreatment in non-small cell lung cancer patients who responded to prior immune-checkpoint inhibitors and had ICI-free intervals (WJOG9616L). Clin Cancer Res.

[19] Bagchi S, Yuan R, Engleman EG (2021). Immune checkpoint inhibitors for the treatment of cancer: clinical impact and mechanisms of response and resistance. Annu Rev Pathol.

[20] Cai Z, Zhan P, Song Y, Liu H, Lv T (2022). Safety and efficacy of retreatment with immune checkpoint inhibitors in non-small cell lung cancer: a systematic review and meta-analysis. Transl Lung Cancer Res.

[21] Haratani K, Hayashi H, Chiba Y (2018). Association of immune-related adverse events with nivolumab efficacy in non-small-cell lung cancer. JAMA Oncol.

[22] Fujisaki T, Watanabe S, Ota T (2021). The prognostic significance of the continuous administration of anti-PD-1 antibody via continuation or rechallenge after the occurrence of immune-related adverse events. Front Oncol.

[23] Santini FC, Rizvi H, Plodkowski AJ (2018). Safety and efficacy of re-treating with immunotherapy after immune-related adverse events in patients with NSCLC. Cancer Immunol Res.

[24] Guo M, VanderWalde AM, Yu X, Vidal GA, Tian GG (2022). Immune checkpoint inhibitor rechallenge safety and efficacy in stage IV non-small cell lung cancer patients after immune-related adverse events. Clin Lung Cancer.

[25] Inno A, Roviello G, Ghidini A (2021). Rechallenge of immune checkpoint inhibitors: a systematic review and meta-analysis. Crit Rev Oncol Hematol.

[26] Garon EB, Rizvi NA, Hui R (2015). Pembrolizumab for the treatment of non–small-cell lung cancer. N Engl J Med.

[27] Topalian SL, Hodi FS, Brahmer JR (2012). Safety, activity, and immune correlates of anti–PD-1 antibody in cancer. N Engl J Med.

[28] Schneider BJ, Naidoo J, Santomasso BD (2021). Management of immune-related adverse events in patients treated with immune checkpoint inhibitor therapy: ASCO guideline update. J Clin Oncol.

[29] Haanen JBAG, Carbonnel F, Robert C (2017). Management of toxicities from immunotherapy: ESMO clinical practice guidelines for diagnosis, treatment and follow-up. Ann Oncol.

[30] Zhao Q, Zhang J, Xu L (2021). Safety and efficacy of the rechallenge of immune checkpoint inhibitors after immune-related adverse events in patients with cancer: a systemic review and meta-analysis. Front Immunol..

